# Multilateral benefit-sharing from digital sequence information will support both science and biodiversity conservation

**DOI:** 10.1038/s41467-022-28594-0

**Published:** 2022-02-23

**Authors:** Amber Hartman Scholz, Jens Freitag, Christopher H. C. Lyal, Rodrigo Sara, Martha Lucia Cepeda, Ibon Cancio, Scarlett Sett, Andrew Lee Hufton, Yemisrach Abebaw, Kailash Bansal, Halima Benbouza, Hamadi Iddi Boga, Sylvain Brisse, Michael W. Bruford, Hayley Clissold, Guy Cochrane, Jonathan A. Coddington, Anne-Caroline Deletoille, Felipe García-Cardona, Michelle Hamer, Raquel Hurtado-Ortiz, Douglas W. Miano, David Nicholson, Guilherme Oliveira, Carlos Ospina Bravo, Fabian Rohden, Ole Seberg, Gernot Segelbacher, Yogesh Shouche, Alejandra Sierra, Ilene Karsch-Mizrachi, Jessica da Silva, Desiree M. Hautea, Manuela da Silva, Mutsuaki Suzuki, Kassahun Tesfaye, Christian Keambou Tiambo, Krystal A. Tolley, Rajeev Varshney, María Mercedes Zambrano, Jörg Overmann

**Affiliations:** 1 Leibniz Institute DSMZ German Collection of Microorganisms and Cell Cultures, Braunschweig, Germany; 2 Leibniz Institute of Plant Genetics and Crop Plant Research (IPK), Seeland, Germany; 3 Natural History Museum, London, UK; 4 One Planet Solutions, Montpellier, France; 5 Universidad de los Andes, Bogotá, Colombia; 6 Plentzia Marine Station (PiE-UPV/EHU), European Marine Biological Resource Centre – Spain (EMBRC-Spain), Plentzia, Spain; 7 Ethiopian Biotechnology Institute, Addis Ababa, Ethiopia; 8 National Academy of Agricultural Science and Global Plant Council, New Delhi, India; 9 National Council of Scientific Research and Technologies (NCSRT), Algiers, Algeria; 10 Ministry of Agriculture, Livestock, Fisheries and Cooperatives, Nairobi, Kenya; 11 Institut Pasteur, Paris, France; 12 School of Biosciences, Cardiff University, Cardiff, UK; 13 Wellcome Sanger Institute, Hinxton, UK; 14 European Molecular Biology Laboratory European Bioinformatics Institute (EMBL-EBI), Hinxton, UK; 15 Global Genome Initiative, Smithsonian National Museum of Natural History, Washington, DC USA; 16 Alexander von Humboldt Biological Resources Research Institute, Bogota, Colombia; 17 South African National Biodiversity Institute, Cape Town, South Africa; 18 University of Nairobi, Nairobi, Kenya; 19 Instituto Tecnologico Vale (ITV), Belem, Brazil; 20 Ministry of Environment and Sustainable Development, Bogota, Colombia; 21 University of Lethbridge, Lethbridge, Canada; 22 Natural History Museum of Denmark, Copenhagen, Denmark; 23 University of Freiburg, Freiburg, Germany; 24 National Centre for Cell Science, Pune, India; 25 Mariano Galvez University, Guatemala City, Guatemala; 26 National Center for Biotechnology Information, National Library of Medicine, National Institutes of Health, Bethesda, MD USA; 27 Centre for Ecological Genomics and Wildlife Conservation, University of Johannesburg, Johannesburg, South Africa; 28 University of the Philippines Los Banos, Laguna, Philippines; 29 Fundação Oswaldo Cruz (FIOCRUZ), Rio de Janeiro, Brazil; 30 National Institute of Genetics, Mishima, Japan; 31 Institute of Biotechnology, Addis Ababa University, Addis Ababa, Ethiopia; 32 Centre for Tropical Livestock Genetics and Health (CTLGH) - International Livestock Research Institute (ILRI), Nairobi, Kenya; 33 Murdoch University, Murdoch, Australia; 34 Corporación CorpoGen, Bogotá, Colombia; 35 Technical University of Braunschweig, Braunschweig, Germany

**Keywords:** Developing world, Policy, Genomics, Genetic databases, Conservation biology

## Abstract

Open access to sequence data is a cornerstone of biology and biodiversity research, but has created tension under the United Nations Convention on Biological Diversity (CBD). Policy decisions could compromise research and development, unless a practical multilateral solution is implemented.

## Abstract (web summary)

Ensuring international benefit-sharing from sequence data without jeopardising open sharing is a major obstacle for the Convention on Biological Diversity and other UN negotiations. Here, the authors propose a solution to address the concerns of both developing countries and life scientists.

Here, we lay out a framework for use of digital sequence information (DSI) that enables fair benefit-sharing, ensures open access to sequence data, strengthens biodiversity conservation and sustainable use, and leverages genomics and bioinformatics for international capacity-building. As Parties to the CBD meet again in-person in the coming months to negotiate the Global Biodiversity Framework, they must apply pragmatic, multilateral solutions to DSI that improve rather than impede global biodiversity targets.

The ability to decode and digitally archive DNA has revolutionized the life sciences and related fields. Sequence data, referred to as digital sequence information (DSI) in policy circles, are key to scientific advancement and technological innovation in fields as diverse as medicine, food security, green energy production, and biodiversity conservation. For example, free and open access to the SARS-CoV-2 viral sequences enabled the rapid development of diagnostic kits and vaccines. Besides its relevance for the common good, DSI is also essential for many commercial applications and offers new perspectives for economic development worldwide. Nonetheless, there are serious concerns about the equitable distribution of these benefits from a global perspective.

Parties to the Convention on Biological Diversity (CBD, https://www.cbd.int/) have recognized that countries have a sovereign right to regulate access to their genetic resources by requiring users to obtain prior informed consent and accept mutually agreed terms that ensure benefit sharing, i.e., that a portion of the advantages or profits derived from use are shared with the providing country^1^. This arrangement is often referred to as simply “ABS” for Access and Benefit-Sharing. These bilateral procedures between countries and users are codified in the CBD’s Nagoya Protocol, which puts forth transactional procedures, compliance mechanisms, and checkpoints that are intended to monitor and ensure benefit-sharing. While well-intentioned, this system has proven to be inefficient and often incurs high transaction costs to obtain the necessary permits^2^.

The rules for accessing DSI produced from these same sovereign genetic resources are, however, generally unclear. DSI is typically held in online open-access databases, where use is disconnected from physical access and accompanying permits. Biodiverse nations, many of which are low- and middle-income countries (LMICs), believe their sovereign rights have been undermined because any potential monetary gains from DSI through commercialization are not shared back to them, as they would be with a genetic resource. Thus DSI is perceived as a loophole that inhibits fair and equitable benefit-sharing. Moreover, the political pressure to close this loophole is high. Multiple authors on this paper have attended online negotiations, in which negotiators have explicitly stated that international benefit-sharing from DSI is a precondition to reach political consensus on the Global Biodiversity Framework. In other words, DSI is a make or break issue for one of the most important decadal environmental deals.

Ironically, according to a recent high-level report^3^, scientists must have open access to DSI to fulfill the aims of the Global Biodiversity Framework (https://www.un.org/sustainabledevelopment/blog/2021/07/a-new-global-framework-for-managing-nature-through-2030-1st-detailed-draft-agreement-debuts/) and the UN Sustainable Development Goals (https://sdgs.un.org/goals).

## What are the current options?

Given these pressures, Parties want options. A range of monetary benefit-sharing options for DSI were recently synthesized by the CBD Secretariat^4^. These range from a bilateral system similar to the CBD and its Nagoya Protocol, with access tightly coupled to benefit sharing, to multilateral mechanisms in which access is facilitated through standardized global rules for benefit-sharing. In March 2020, Laird et al.^5^, made a compelling case for multilateralism, and called for the scientific community to work together to develop policy options, but left unanswered what a policy framework could look like.

**Fig. 1:**
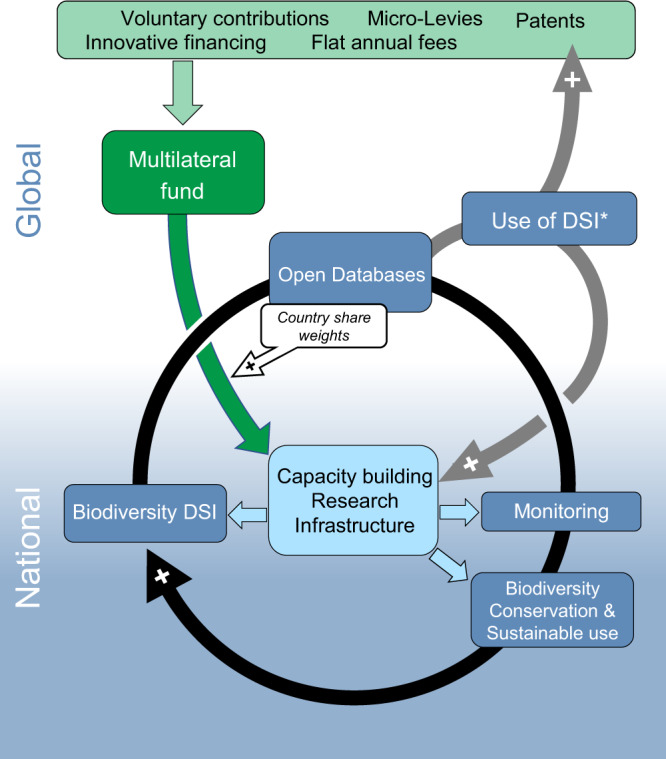
Multilateral funding model for DSI benefit-sharing. Open access to DSI in databases (center blue box) is de-coupled from benefit-sharing (light green box). The multilateral fund (dark green box) can collect funds from micro-levies, voluntary contributions, innovative finance, or from patent royalties. Funding on a national level (dark green arrow) is weighted by the amount of sequences deposited from an individual LMIC, and together with the substantial non-monetary benefit-sharing from DSI (e.g., through international collaborations; lower gray arrow) enables capacity building (especially in the area of genomics and bioinformatics) as well as the build-up of a corresponding research infrastructure. Capacity building improves monitoring, conservation, and sustainable use of biodiversity (dark blue boxes), creating a positive feedback loop that leads to a continuous increase in knowledge generation on biodiversity. Simultaneously, greater availability of DSI creates additional positive feedback loops for generating non-monetary and monetary benefits (gray arrows). The asterisk (*) emphasizes that the use of DSI, and hence the benefits generated, largely depend on the use of DSI originating from several different sources (i.e., countries).

We respond to this call and provide a concrete framework for how DSI benefit-sharing could work (Fig. 1). The authors are members of the *DSI Scientific Network*, a group of scientists from different countries and economic settings that share convergent points of view in the DSI debate. We note that every country provides and uses DSI and, thus, everyone has something to lose and gain^6^. Here, we map out a multilateral framework for DSI that will support biodiversity monitoring and conservation, maintain open access, improve the scientific record, share both non-monetary and monetary benefits, and enable green growth.

## The DSI policy option that will not work

The core infrastructure for storing sequence data, the International Nucleotide Sequence Database Collaboration (INSDC) contains 228 million annotated sequences (1.5 billion reads, see https://www.ncbi.nlm.nih.gov/genbank/statistics/). The dataset is downloaded partially or completely 34 million times per year, used by more than 10–15 million unique users, and 99.5% of the 750 downstream sequence databases that pull and push DSI through the scientific ecosystem directly rely on the INSDC system^7^. The data are linked to a further 1200 databases and hundreds of thousands of publications that, on average, cite 44 sequences per publication^6^. Clearly, a bilateral system, modelled on the principles of the Nagoya Protocol, that required permission between the end-user and the country of origin for every sequence and user transaction, would be prohibitively complex, affect data interoperability, and be ill-suited for generating knowledge.

The DSI statistics above make clear that a human-based system would not work. Technological approaches to track and trace DSI usage would be expensive, complex, and could have a significant environmental footprint. Most importantly, a loss of open access would adversely impact the analytical capacities, data infrastructures, and academic systems in LMICs that rely on these open data infrastructures^2,5,8–10^ and make collaboration with researchers based in countries that enforce DSI use restrictions very unattractive.

A bilateral DSI system would also inadvertently create a competitive marketplace that would perversely incentivize the use of DSI from countries where no restrictions exist (e.g., USA, Germany) leading to DSI “forum shopping.” Ironically, DSI from LMICs that are needed most for global monitoring and protection of biodiversity would often be avoided which, in turn, would severely impede progress on the Global Biodiversity Framework.

## A win-win multilateral option for DSI

A benefit-sharing framework for DSI clearly needs to be multilateral and should address five fundamental objectives:

1. *Open access.* Any future benefit-sharing system must guarantee open data access, which is required to be able to use and understand DSI. Only open access enables efficient and broad scale knowledge generation and capacity building. The existing core DSI infrastructure already fulfils these requirements.
2. *Simplicity.* The DSI data ecosystem is highly complex, even for expert users. For benefit-sharing to happen, the policy framework must be simple. If a complex regulatory layer is added on top of a complex technical system, it is doomed to fail.
3. *Harmonize.* The DSI dilemma is an opportunity to learn from current inefficiencies in ABS and minimize transaction costs. Furthermore, as DSI is being discussed in multiple international fora, DSI users need a harmonized framework to address benefit-sharing.
4. *Biodiversity.* Any mechanism needs to effectively support biodiversity conservation and sustainable use (the first two objectives of the CBD). The framework should incentivize and reward biodiversity knowledge generation, and fill in the blank spots on the world map of biodiversity^11^.
5. *Fairness.* The framework should treat all users and providers fairly and create a level playing field by facilitating both access and compliance evenly across the globe. A multilateral system could nevertheless include opportunities for country-specific recognition and differentiated distribution of funds.

In Fig. 1, we propose a multilateral DSI benefit-sharing framework where access to DSI is “decoupled” from benefit-sharing from DSI. Benefit-sharing is ensured by mechanisms that do not limit access to DSI. This is a fundamental shift away from traditional control-oriented ABS to a new idea of OA (open access) and BS (benefit-sharing). This is necessary to protect the many benefits of openness and recognize that benefit-sharing can be accomplished without dramatically altering real-world access. New monetary mechanisms can be put into place upstream of DSI generation (e.g., a micro-levy on DSI-generation reagents and disposables), downstream of DSI use (e.g., a user fee on bio-based products), and/or outside the DSI life cycle (e.g., payment from high-income nation international development funds).

To achieve simplicity, benefit-sharing must be based upon the entire global DSI dataset and not on individual sequences. This reflects the science: analysis of sequences can only be done by comparing them to all other DSI and, thus, have zero value in isolation. Fundamental bioinformatic techniques, like sequence alignment, search, and annotation, are founded on this comparative principle. We caution, however, against paywalls which, although simple and understandable, would not qualify as open access and would fundamentally break the interoperability of data in the current DSI ecosystem. In our model, the DSI data ecosystem itself would remain virtually the same for the user and would need only modest behind-the-scenes adaptation to generate funds.

To incentivize the generation of biodiversity data, funds would be distributed via project-based applications based on a country’s development status and their DSI contribution to the global dataset. Those LMICs that contribute more DSI to the global dataset (by providing access to genetic resources), would receive comparatively more funds. This would create an incentive to illuminate biodiversity blind spots and build up in-country expertise, thereby creating a positive feedback loop. Notably, such a system could also be expanded to other international fora that are debating DSI such as the International Treaty on Plant Genetic Resources for Food and Agriculture, the World Health Organization, and the UN Convention on the Law of the Sea potentially yielding a “harmonized” solution.

## Increased responsibilities for scientists and databases

For this system to work, the scientific community needs to improve practice with regard to recording the country of origin for DSI and correct legacy problems. At present, only 16% of sequences in the INSDC have country of origin information associated with them; 44% of sequences without country data could and should have had country information provided by the submitting scientists^7^. Geographical information is not only important for policymakers, but it is also useful scientific data that should be reported as a matter of scientific integrity and the FAIR principles, which place a strong emphasis on the importance of reliable “provenance” metadata^12^. Importantly, the INSDC recently announced a new policy (https://www.insdc.org/spatio-temporal-annotation-policy-18-11-2021) requiring spatio-temporal information demonstrating their commitment to scientific transparency and openness to change.

Challenges still remain. For example, sequences that are listed in a patent application often have country information listed in the patent application. Although these same sequences are automatically submitted to the INSDC by patent offices, the country information is not transferred. This remains a significant loss of transparency given the fact that patent-associated DSI represents 20% of the INSDC dataset^7^.

Nevertheless, scientific databases are not regulatory entities. They are scientific infrastructures with a public mission. In our view, they can play a supportive role in the policy process by providing data to policymakers. As part of the Global Biodiversity Framework, DSI-holding databases could be called upon to coordinate and deliver “country reports”—global analysis of DSI submissions, user trends, capacity building and advancement on DSI provenance transparency (see above). These data could be linked back to the benefit-sharing calculation. Given that the INSDC is an American, European, and Japanese collaboration (all high-income countries and the US is a CBD observer), it would likely be advisable to work in close collaboration with a more diverse group of databases. Here, the increasingly active Global Biodata Coalition could play a leading role (https://globalbiodata.org/).

There remain unanswered questions such as how to ensure fairness to indigenous people and local communities in a multilateral system. This could be encouraged via a new data tag for indigenous people and local communities, for example^13^ and simply making IPLCs directly eligible to receive monetary funds. It would also be counter-productive if users began to dump low-quality or repetitive DSI into the databases to “game” the system and artificially increase a country’s DSI proportion in the database. Here standards and internal quality checks would be essential and are actually realizable. Finally, economic modelling must be conducted to determine which of the monetary mechanisms listed above are most likely to be cost-effective, financially productive, and to minimize administrative costs.

## DSI capacity-building enables “leapfrogging” towards the bioeconomy

While all countries use and produce DSI, there remain significant inequalities. Practical issues ranging from more expensive access to molecular biological reagents, slower internet bandwidth that limits high-throughput analyses, financial limitations for research funding, limited bioinformatics training and career development opportunities, as well as brain drain, routinely limit those of us working in LMICs. Thus, any DSI benefit-sharing framework must support technical capacity building focused on genomics and bioinformatics. The goal should be to facilitate a “leapfrog” effect in which LMIC scientists are trained to exploit DSI even while inequalities in high-tech sequencing or laboratory infrastructure including technology transfer are still being addressed. With advances in cloud computing, open-source software, and open access DSI databases, the gaps are easier to fill than ever before if strategic investments are made.

Science-focused capacity development within the CBD must be aimed both at conservation and building up the bioeconomy through sustainable use of bioresources. Local scientists and regional or national science academies should be involved in agenda-setting. Matchmaking platforms should be established that connect scientists across the globe and build up human capital enabling sustainable development.

## Call to action

Decisions made at the Geneva meetings in March 2022 and, ultimately, at the 15th Conference of the Parties to the CBD later in 2022, will affect DSI practitioners for a generation to come. The crux of the decision will be whether governments want “control” over DSI through a bilateral system or whether they see the opportunity for greater scientific advancement and ultimately greater monetary value if they choose openness through a multilateral system.

Given the importance of DSI for the scientific community, we call for policymakers to affirm multilateralism at the COP15 meeting and de-couple access to DSI from benefit-sharing—to break the “ABS” acronym into “OA” and “BS” where open access, benefit-sharing, capacity building, and biodiversity conservation thrive. We encourage policymakers to engage with their national DSI practitioners and with our Network to work together towards a thoughtful, workable and informed compromise.

## References

[1] Schroeder D (2007). Benefit sharing: it’s time for a definition. J. Med. Ethics.

[2] Prathapan KD (2018). When the cure kills—CBD limits biodiversity research. Science.

[3] IPBES. Global assessment report on biodiversity and ecosystem services of the Intergovernmental Science-Policy Platform on Biodiversity and Ecosystem Services. *Zenodo*10.5281/zenodo.5517154 (2019).

[4] Policy options for Access and Benefit Sharing and Digital Sequence Information. *Secretariat of the Convention on Biological Diversity* https://www.cbd.int/abs/DSI-webinar/DSIPolicyOptions2021.pdf (2021).

[5] Laird S (2020). Rethink the expansion of access and benefit sharing. Science.

[6] Scholz AH (2021). Myth-busting the provider-user relationship for digital sequence information. GigaScience.

[7] Rohden, F., Huang, S., Dröge, G. & Scholz, A. H. *Combined study on digital sequence information in public and private databases and traceability*. Report No. CBD/DSI/AHTEG/2020/1/4 https://www.cbd.int/doc/c/1f8f/d793/57cb114ca40cb6468f479584/dsi-ahteg-2020-01-04-en.pdf (2020).

[8] Bockmann FA (2018). Brazil’s government attacks biodiversity. Science.

[9] Ribeiro C (2018). How ownership rights over microorganisms affect infectious disease control and innovation: A root-cause analysis of barriers to data sharing as experienced by key stakeholders. PLoS ONE.

[10] Rourke M, Eccleston-Turner M, Phelan A, Gostin L (2020). Policy opportunities to enhance sharing for pandemic research. Science.

[11] Tydecks L, Jeschke JM, Wolf M, Singer G, Tockner K (2018). Spatial and topical imbalances in biodiversity research. PLoS ONE.

[12] Wilkinson MD (2016). The FAIR Guiding Principles for scientific data management and stewardship. Sci. Data.

[13] Liggins L, Hudson M, Anderson J (2021). Creating space for Indigenous perspectives on access and benefit‐sharing: Encouraging researcher use of the Local Contexts Notices. Mol. Ecol..

